# Porewater microbial community dynamics act as an indicator of northern peatland ecosystem change in response to climate drivers

**DOI:** 10.1093/ismeco/ycag164

**Published:** 2026-06-15

**Authors:** Madison Green, Tianze Song, Caitlin Petro, Jose L Rolando, Katherine Duchesneau, Josephine Treitz, Rachel M Wilson, Jeffrey P Chanton, Joel E Kostka

**Affiliations:** School of Biological Sciences and School of Earth and Atmospheric Sciences, Center for Microbial Dynamics and Infection, Georgia Institute of Technology, Atlanta, GA 30332, United States; School of Biological Sciences and School of Earth and Atmospheric Sciences, Center for Microbial Dynamics and Infection, Georgia Institute of Technology, Atlanta, GA 30332, United States; Institute for Sustainability, Energy, and Environment, University of Illinois Urbana-Champaign, Urbana, IL 61801, United States; School of Biological Sciences and School of Earth and Atmospheric Sciences, Center for Microbial Dynamics and Infection, Georgia Institute of Technology, Atlanta, GA 30332, United States; School of Biological Sciences and School of Earth and Atmospheric Sciences, Center for Microbial Dynamics and Infection, Georgia Institute of Technology, Atlanta, GA 30332, United States; Microbiology and Cell Science Department, Institute of Food and Agricultural Sciences, Fort Lauderdale Research and Education Center, University of Florida, Davie, FL 33314, United States; School of Biological Sciences and School of Earth and Atmospheric Sciences, Center for Microbial Dynamics and Infection, Georgia Institute of Technology, Atlanta, GA 30332, United States; School of Integrative Plant Science, Cornell University, Ithaca, NY 14853, United States; School of Biological Sciences and School of Earth and Atmospheric Sciences, Center for Microbial Dynamics and Infection, Georgia Institute of Technology, Atlanta, GA 30332, United States; Department of Chemistry and Biology, University of Wuppertal, 42097 Wuppertal, Germany; Department of Earth, Ocean, and Atmospheric Science, Florida State University, Tallahassee, FL 32306, United States; Department of Earth, Ocean, and Atmospheric Science, Florida State University, Tallahassee, FL 32306, United States; School of Biological Sciences and School of Earth and Atmospheric Sciences, Center for Microbial Dynamics and Infection, Georgia Institute of Technology, Atlanta, GA 30332, United States

**Keywords:** peatland, climate change, microbial diversity, carbon cycling, methanogenesis, methanotrophy

## Abstract

Northern peatlands store approximately one-third of all terrestrial carbon, making their belowground microbial communities key regulators of peatland carbon cycling and climate feedbacks. We examined how climate drivers alter microbial community composition across peat and porewater habitats over a 0–200 cm depth profile by leveraging the SPRUCE (Spruce and Peatland Responses Under Changing Environments) experiment. SPRUCE uses whole-ecosystem warming to simulate temperature increases from 0°C to +9°C above ambient as well as elevated carbon dioxide (CO₂) treatments. Through small subunit ribosomal ribonucleic acid amplicon sequencing, we found significant differences in taxonomic diversity, abundance, and community composition between attached (peat) and free-living (porewater) microbial assemblages. Porewater communities exhibited significantly higher microbial diversity, lower abundance, and were more responsive to ecosystem-level manipulations (warming and CO₂) than their peat counterparts. The relative abundance of putative methane-cycling microorganisms, methanogens, and methanotrophs was comparable in surface peat and porewater, but at deeper depths, methanotrophs were far more abundant in porewater. Additionally, warming more strongly stimulated putative methanotrophs than methanogens, particularly in porewater, resulting in a more abundant methanotrophic community across all depths. Our results show that peat and porewater habitats harbor distinct microbial communities that respond differently to climate change drivers. The distinct nature of porewater communities, particularly the observed dynamics of methanotrophs, underscores their role in peatland carbon cycling and highlights their potential as sensitive indicators of environmental change, with significant implications for peatland restoration and carbon management in a changing climate.

## Introduction

Northern peatlands play a well-established role in the global carbon cycle: despite occupying ~3% of total landmass, they store 30%–50% of terrestrial carbon [1–4] and act as both a net carbon dioxide (CO_2_) sink and a net methane (CH_4_) source [5–7]. Because heterotrophic microbial communities mediate carbon sequestration and greenhouse gas emissions, climate-change studies have focused on these communities, typically in the cold, acidic, waterlogged peat matrix that defines the ecosystem [8]. Yet, continuous saturation also creates distinct niches in interstitial porewater, and the free-living communities residing there, along with their roles in the carbon cycle and their responses to climate drivers, remain largely uncharacterized.

Northern peatlands accumulate carbon through an imbalance between plant uptake and microbial decomposition [9, 10]. Rising temperatures threaten this balance by simultaneously extending the growing season, accelerating decomposition, and increasing evapotranspiration [2, 11–13], while vascular plant proliferation alters the quality and availability of dissolved organic matter (DOM) [14]. These coupled plant–microbial responses complicate predictions of peatland carbon emissions under environmental change, making mechanistic understanding of belowground carbon turnover critical [15, 16]. Although porewater DOM represents a smaller pool than solid peat, it drives microbial CO_2_ and CH_4_ production [17–19] and can both enhance and inhibit decomposition of soil organic matter [20–23].

Porewater microbial communities may offer a practical and sensitive readout of climate-driven change: porewater is relatively homogeneous, sampled non-destructively, and may capture more dynamic responses than bulk peat. Yet integrated field studies of porewater and peat communities under climate manipulation remain rare. We addressed this gap using the Spruce and Peatland Responses Under Changing Environments (SPRUCE) experiment, a whole-ecosystem warming study in a northern Minnesota peatland [24], in which enclosures simulate warming from 0 to +9°C under ambient and elevated atmospheric CO_2_. We characterized composition and diversity in both habitats by small subunit ribosomal ribonucleic acid (SSU rRNA) gene amplicon sequencing and quantitative polymerase chain reaction (qPCR), examining responses to warming and elevated CO_2_ over multiple years. Porewater samples span 2017–2021, and peat samples 2017–2019. Because deep peat heating began in June 2014, ecosystem warming in 2015, and CO_2_ treatment in June 2016, this window captures responses well beyond the initial transient phase.

Prior SPRUCE work has either reported stable peat microbial composition under warming from long-term monitoring [23, 25, 26] or used incubations to probe porewater constituents rather than in situ dynamics [23], leaving the contribution of porewater to the peatland carbon budget unresolved. Yet warming has been shown to increase surface CO₂ and CH₄ emissions despite apparent stability of peat communities [19, 27], suggesting that peat-only sampling provides an incomplete view of microbial responses.

We hypothesize that (i) porewater and peat communities differ owing to contrasting substrate availability (DOM, oxygen, CH_4_) in free-living versus attached habitats; (ii) porewater communities are more sensitive to environmental change because of less-limited diffusion of plant-derived compounds and the strong influence of warming on plant community composition; and (iii) climate drivers will produce substantial compositional shifts via differential adaptive responses among taxa.

## Materials and methods

### Study site and experimental design

The SPRUCE experiment is located in the S1 bog of the Marcell Experimental Forest, near Grand Rapids, MN, USA (47°30.476′N, 93°27.162′W), an acidic, ombrotrophic peatland described in detail elsewhere [27–30]. The site is a whole-ecosystem climate manipulation comprising ten open-top enclosures and two ambient control plots [31, 32]. Whole-ecosystem warming is maintained at +0, +2.25, +4.5, +6.75, and +9°C above ambient via aboveground air heating combined with deep peat heating [24]. Each warming level is crossed with ambient or elevated CO_2_ (+500 ppm, targeting ~900 ppm) chosen as an upper end-of-century projection [24, 33]. Deep peat heating began in June 2014, ecosystem warming in 2015, and CO_2_ treatments in June 2016. Additional infrastructure details are provided in the Supplemental methods.

### Sample collection

Porewater was collected from permanently installed polyvinyl chloride (PVC) piezometers at depths of 25, 50, 100, and 200 cm below the peat hollow surface during the peak growing season (late July–early August) from 2017–2021. Piezometers were pumped dry, recharged for 12 hours, sampled by peristaltic pump, filtered through 0.2 μm sterile filters within 1 hour of collection, and stored on dry ice. Peat was collected by Russian coring across the 0–200 cm profile during annual coring events (August 2017–2019), sectioned by depth, homogenized, and flash-frozen. Paired peat and porewater samples were collected from the same enclosure within the same week, shipped on dry ice to Georgia Tech, and stored at −80°C.

### Quantitative polymerase chain reaction

qPCR with universal bacterial and archaeal SSU rRNA primers was used to estimate microbial abundance in 96 paired peat and porewater samples collected in 2019 (all five temperature treatments × four depths × both CO₂ treatments) following established protocols [23, 34, 35]. Gene abundances were normalized to 1 ml of porewater or to wet peat; primer sequences [36, 37] and the volumetric correction [38] are given in the Supplemental methods.

### Deoxyribonucleic acid extraction, amplification, and sequencing

Deoxyribonucleic acid (DNA) was extracted using the Qiagen PowerSoil DNA Kit. The 16S rRNA gene V4 region was amplified and sequenced on an Illumina MiSeq2000 (paired-end 250 bp); 2017–2018 samples were processed at the University of Illinois at Chicago, and 2019–2021 samples at Georgia Tech using updated primer sets [39, 40]. Primer sequences are listed in Supplemental methods.

### Sequence processing

Raw sequences were trimmed with Cutadapt, amplicon sequence variants (ASVs) inferred with DADA2 [41], and taxonomy assigned against SILVA v138.1 [42]. Chloroplast, mitochondrial, and unclassified-phylum sequences were removed. Sequences were aligned with DECIPHER and phylogenies built with FastTree [43]. Putative methanotroph and methanogen functions were inferred from genus-level homology to described prokaryotic species (Supplemental Table 1). Sequences are deposited under NCBI BioProject PRJNA1327265.

### Statistical analysis

Samples were rarefied to 14 419 reads, and alpha diversity (observed ASVs and Shannon) was calculated using phyloseq and picante [44, 45]. For primary analyses, ASV counts were normalized by cumulative sum scaling (CSS); relative abundances were derived by within-sample proportional transformation after agglomeration at the genus or phylum level.

**Figure 1:**
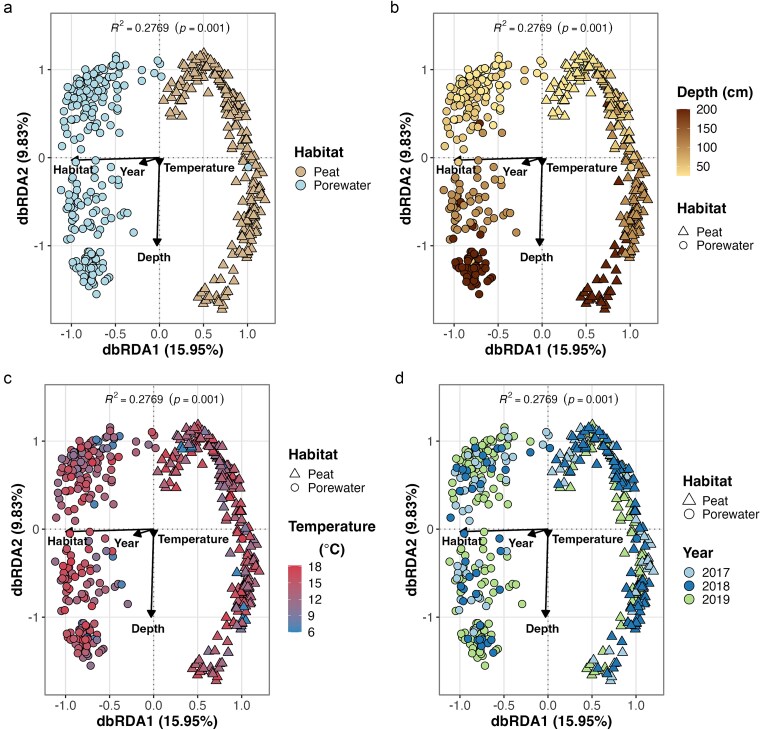
db-RDA of peat and porewater community compositions in relation to environmental factors. Ordination uses Bray–Curtis dissimilarity matrix among CSS-normalized 16S rRNA gene ASV communities from peat and porewater samples collected at four depths (25, 50, 100, 200 cm) across three years (2017, 2018, 2019) and all treatment enclosures. Panels are colored by (a) habitat (peat vs. porewater), (b) depth (25–200 cm), (c) measured temperature at 50 cm depth, and (d) sampling year (2017–2019). Biplot arrows show the directions and relative magnitudes of the constrained environmental variables (habitat, depth, temperature, and year). The overall model R^2^ and permutational *P*-value are annotated on each panel. Triangles represent peat samples; circles indicate porewater samples.

Community composition was analyzed by distance-based redundancy analysis (dbRDA, vegan) on Bray–Curtis dissimilarities [44, 46]. The primary model (Fig. 1) included habitat, depth, sampling year, and measured temperature at 50 cm; a secondary model (Supplemental Fig. 4) added porewater CH_4_, CO_2_, and dissolved organic carbon (DOC). Variance partitioning (varpart(), vegan) on Hellinger-transformed abundances quantified unique contributions of habitat, depth, year, and temperature, with a second partitioning for DOC, temperature, depth, and dissolved gasses run on the full dataset and separately by habitat (Supplemental Table 2). Unique contributions were tested by partial dbRDA (999 permutations).

Differential abundance was assessed using MaAsLin2 [47] across 64 models, each combining habitat, depth, taxonomic rank, and a fixed effect (temperature or CO_2_), with year as a random effect. Relationships between CH_4_-cycling taxon abundances and environmental predictors were evaluated by OLS linear regression (Fig. 5, Supplemental Fig. 7). Full model specifications, thresholds, and permutation schemes are detailed in the Supplemental methods.

### Environmental data

Porewater chemistry (DOC, pH, NH₄^+^, NO₃^−^) was obtained from the Griffiths dataset [48] and dissolved CH_4_ and CO_2_ from the Wilson metabolome dataset [49]. Variables were averaged monthly at the plot × depth level and merged with 16S metadata by plot, depth, year, and month.

## Results

### Partitioning of microbial diversity by habitat

Alpha diversity responses to warming were habitat-specific (Supplemental Fig. 1). In peat, neither richness (observed ASVs) nor Shannon diversity changed significantly with temperature at any depth (all *P* > .05), indicating a compositional resistance to warming over the 3 years included in the analysis. In porewater, richness declined significantly with temperature at all four depths (25 cm: R^2^ = 0.15, *P* = .005; 50 cm: R^2^ = 0.21, *P* < .001; 100 cm: R^2^ = 0.13, *P* = .008; 200 cm: R^2^ = 0.10, *P* = .023). Shannon diversity also declined with temperature at 25 cm (R^2^ = 0.24, *P* < .001) and 50 cm (R^2^ = 0.32, *P* < .001), but not at 100 or 200 cm (*P* > .05).

Total bacterial and archaeal 16S rRNA gene copy numbers responded to warming in a manner that depended on depth and habitat (Supplemental Fig. 2). In peat, bacterial abundance was most strongly predicted by temperature at 25 cm (R^2^ = 0.72, *P* = .002), with a weaker but still significant relationship at 200 cm (R^2^ = 0.43, *P* = .040), while no significant trend was observed at 50 or 100 cm. In contrast, archaeal abundance in peat responded to warming only at 50 cm (R^2^ = 0.52, *P* = .028), with no significant temperature effect at other depths. The bacterial-to-archaeal ratio in peat was not significantly associated with temperature at any depth. In porewater, bacterial gene copy numbers increased with warming at 25 cm depth (R^2^ = 0.52, *P* = .018), but showed no significant response at deeper horizons. Archaeal abundance in porewater was not significantly related to temperature at any depth. Notably, the bacterial-to-archaeal ratio in porewater was significantly predicted by temperature at 100 cm (R^2^ = 0.48, *P* = .027), indicating a warming-related shift in the relative contributions of these domains at depth, even when there were no significant individual responses (Supplemental Fig. 2).

### Peat and porewater have distinct microbial communities

Amplicon sequencing generated 28.5 million high-quality sequences across 812 samples, yielding 33 007 ASVs for beta-diversity analysis. A dbRDA was conducted to evaluate the impact of four key environmental factors on microbial community composition: habitat, sampling year, depth, and temperature treatment (Fig. 1). When all four environmental factors were included, the model explained 27.4% (adj. R^2^) of the total community variation (Fig. 1). Samples segregated most strongly by habitat and depth, consistent with their dominant roles as independent predictors: habitat (peat vs. porewater) uniquely accounted for 15.2% of variation (partial dbRDA, F = 88.5, *P* = .001) and depth accounted for 10.0% (F = 58.5, *P* = .001). Temperature treatment and year were each statistically significant but explained substantially less variation independently (0.6%, F = 4.5, *P* = .001, and 0.8%, F = 5.8, *P* = .001, respectively). The shared fraction among all predictors was negligible (0.8%), indicating that these variables structure community composition along largely orthogonal axes.

**Figure 2:**
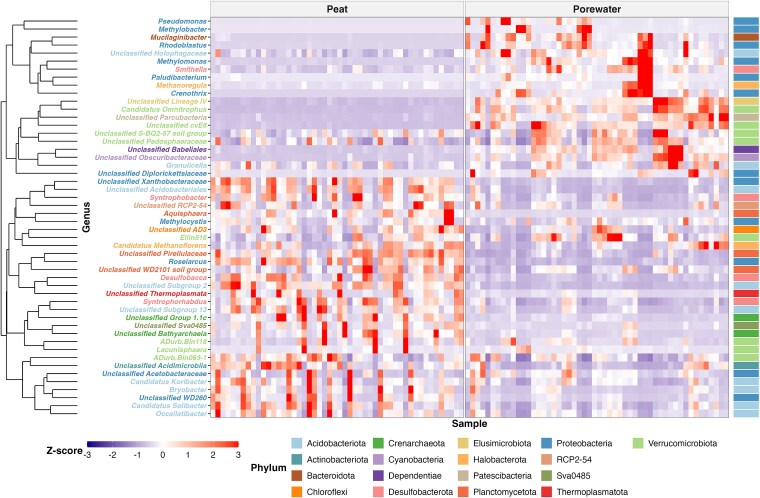
Relative abundance heatmap of the 50 most abundant genera in peat and porewater communities under ambient treatment conditions. Z-score-normalized relative abundances of these genera across samples from three years (2017, 2018, 2019), four depths (25, 50, 100, 200 cm), and only ambient treatment enclosures. Genera are ordered by hierarchical clustering, shown in the dendrogram on the left. Genus names are colored by phylum affiliation (see the phylum annotation bar on the right). Red indicates high abundance; blue indicates low abundance.

To distinguish habitat-intrinsic differences in community composition from effects of the experimental treatment, we limited this analysis to only the ambient (unwarmed, ambient CO_2_) plots (Fig. 2). By excluding treatment enclosures, we aimed to demonstrate that peat and porewater contain fundamentally different microbial communities before examining how treatments affect them. The relative abundances of the 50 most abundant genera were normalized to Z-scores within each genus, so that a value of zero indicates the average abundance across samples for that genus, and a positive or negative value indicates above- or below-average abundance, respectively (Fig. 2). Z-score normalization facilitated fair visual comparisons across genera with widely varying absolute abundances, highlighting compositional patterns rather than the dominance of highly abundant taxa. Community compositions in peat and porewater were clearly distinct, with only 22.8% of ASVs shared between habitats; 63.5% of ASVs were unique to porewater and 13.7% unique to peat, confirming that these environments function as largely separate microbial niches independent of the experimental treatment.

### Porewater communities are more sensitive to climate drivers than peat

**Figure 3:**
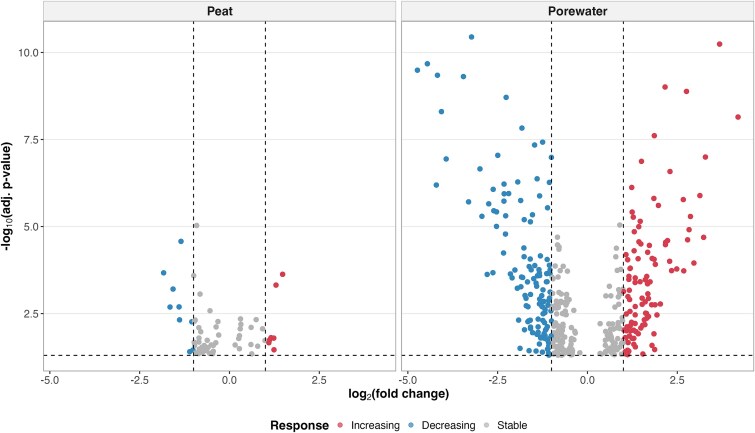
Volcano plots showing differentially abundant genera in response to experimental warming. Log_2_ fold change versus −log_10_(adjusted *P*-value) for genera that significantly change with measured temperature at 50 cm depth, identified by MaAsLin2 (*P* < .05) in peat (left panel) and porewater (right panel) communities. Samples include four depths (25, 50, 100, 200 cm), three years (2017, 2018, 2019), and all treatment enclosures. Red points denote genera with a significant positive response to warming (log₂ fold change ≥1); blue points denote genera with a significant negative response (log₂ fold change ≤ −1); gray points denote non-significant or low-magnitude changes. Dashed lines mark fold change (±1) and significance (*P* = .05) thresholds.

We used MaAsLin2 to identify ASVs significantly associated with each treatment across the full depth profile (peat and porewater) for 2017–2019. Porewater harbored more temperature-responsive ASVs than peat at every depth, contributing 74%, 90%, 82%, and 100% of responsive ASVs at 25, 50, 100, and 200 cm (Supplemental Fig. 3), a disparity not explained by baseline alpha diversity. CO_2_ associations were similarly porewater-dominated (67%–100% at 50–200 cm) but fewer overall, with no significant associations at 25 cm in either habitat. This analysis was also used to generate volcano plots (Fig. 3) illustrating the magnitude and direction of temperature associations: at *P* < .05 and |log₂ fold change| ≥ 1, we identified 179 temperature-associated ASVs in porewater and 16 in peat.

Variance partitioning with DOC, depth, temperature, and dissolved CH_4_ and CO_2_ as predictor sets (Supplemental Fig. 4, Supplemental Table 2) showed that all predictors significantly influenced community composition (*P* < .001, 999 permutations), together explaining 12.9% of variation (adj. R^2^), with depth contributing the largest unique fraction (5.8%), followed by DOC and dissolved gasses (2.5% each) and temperature (2.2%). Habitats diverged sharply: peat was more constrained overall (24.96% vs. 19.11%), with depth (13.4%) and DOC (7.2%) dominant, and temperature explaining only 1.1% uniquely. In porewater, depth remained strongest (7.5%), but temperature emerged as the second-largest fraction (4.7%), exceeding DOC (3.2%) and dissolved gasses (3.8%). DOC was a significant independent predictor in both habitats (peat: F = 17.6, *P* = .001; porewater: F = 11.9, *P* = .001).

**Figure 4:**
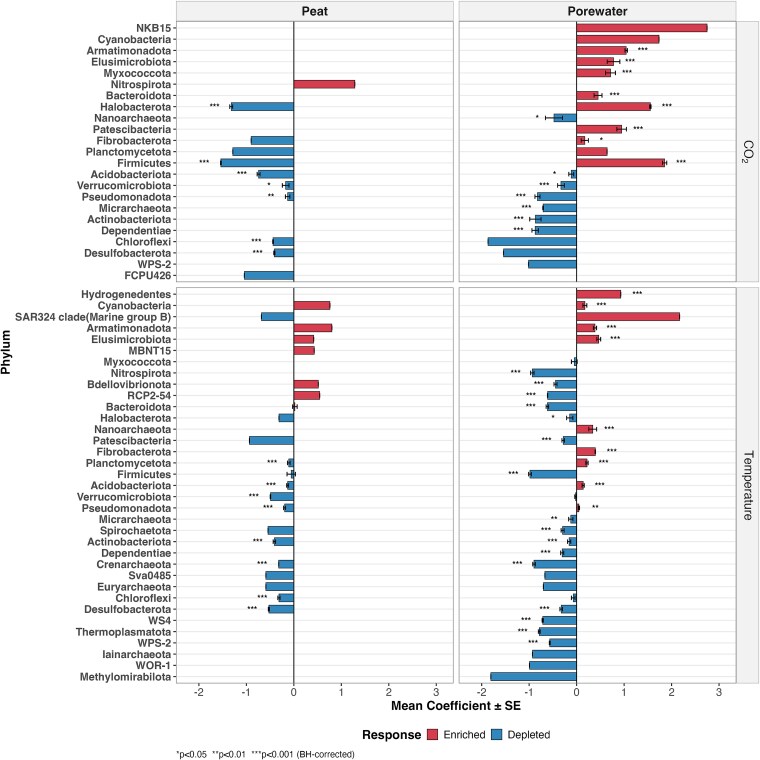
Phylum-level response to temperature and elevated CO₂ in peat and porewater samples. Mean MaAsLin2 coefficients (±SE) of genera aggregated to the phylum level for measured temperature at 50 cm depth (bottom row) and CO₂ (top row) effects in peat (left column) and porewater (right column) communities across three years (2017, 2018, 2019) at four depths (25, 50, 100, 200 cm) and all treatment enclosures. Red bars (positive coefficients) indicate enrichment with increasing temperature or elevated CO₂; blue bars (negative coefficients) indicate depletion. Significance stars indicate BH-corrected p-values from one-sample t-tests of coefficients against zero (*P < .05, **P < .01, ***P < .001).

Phylum-level MaAsLin2 mixed models within each habitat (Fig. 4) identified 27 phyla responsive to temperature and 17 to CO_2_ (*P* < .05), with the magnitude and direction strongly dependent on habitat. In peat, CO_2_ treatments reduced relative abundance across seven phyla, and temperature treatments across eight phyla, with no significant enrichments. Porewater showed broader, bidirectional responses with CO_2_ effects spanning 15 phyla and often reversing those in peat, while temperature responses were predominantly negative but included enrichments absent from peat. Archaea were broadly depleted by warming in both habitats.

### Porewater methane cycling taxa respond to warming

Porewater CH_4_-cycling taxa are key mediators of greenhouse gas dynamics in peatlands. Warming has been shown to stimulate methanogenesis, thereby increasing CH_4_ production relative to CO_2_ [19, 30]. To better understand the microbial drivers of these processes, putative functional roles were inferred from genus-level homology to described prokaryotic species (Supplemental Table 1).

Analysis of putative CH_4_-cycling taxa revealed striking habitat- and depth-dependent distributional differences between methanotrophs and methanogens (Supplemental Fig. 5). Methanotrophs were significantly more abundant in porewater below the surface (50–200 cm; *P* < .0001), with relative abundance increasing with depth, whereas peat methanotrophs peaked at 50 cm and declined with depth. Methanogens showed the opposite pattern: more abundant in shallow porewater (25–50 cm) but increasing with depth in peat, peaking at 200 cm.

Across all samples, we identified 302 methanotrophic ASVs spanning 18 genera and 148 methanogenic ASVs across 15 genera. Genus-level composition highlighted strong habitat differentiation within both guilds (Supplemental Fig. 6). Among methanotrophs, *Methylocystis* dominated in peat but was also abundant in porewater, peaking at 100 cm. In contrast, *Methylobacter* and *Crenothrix* were effectively absent from peat but increased sharply with depth in porewater, suggesting niche partitioning along redox gradients. *Methylomonas* was consistently abundant across all porewater depths despite being nearly absent from peat below 25 cm, making it among the most habitat-differentiated genera in the dataset. Among methanogens, Ca. *Methanoflorens* dominated in peat with a mid-depth peak, while in porewater it was most abundant near the surface and declined sharply at depth, a reversal not observed in peat. *Methanoregula* increased with depth in both habitats, but far more steeply in porewater, while unclassified *Methanomicrobiaceae* was largely restricted to deep peat.

**Figure 5:**
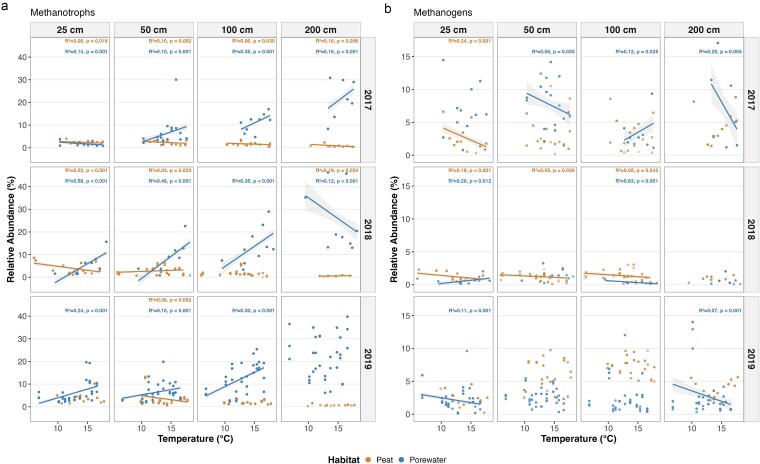
Putative methane-cycling taxa temperature response by sampling year and depth. Total relative abundance of putative (a) methanotrophs and (b) methanogens is plotted against measured temperature at 50 cm depth. Samples span 3 years (2017, 2018, 2019), four depths (25, 50, 200, 200 cm), all treatment enclosures, and are faceted by depth (column) and year (rows). Points are colored by habitat: blue for porewater, tan for peat. Linear regression trendlines with 95% confidence intervals are shown only for significant models (*P* < .05), with corresponding R^2^ and *P*-values annotated. Models were fit independently for each group × habitat × depth × year combination; only combinations with *n* ≥ 5 observations are included.

To evaluate temperature responses within CH_4_-cycling communities, we regressed relative abundance on measured temperature for each combination of habitat, depth, and year using OLS (Fig. 5). Methanotroph responses were strongly habitat-dependent: porewater methanotroph abundance increased significantly with temperature in 9 of 12 models (R^2^ = 0.10–0.59), while peat methanotroph responses were predominantly negative (6 of 8 significant models). Methanogens declined with warming across both habitats, though with weaker overall temperature sensitivity than methanotrophs (13 of 24 models non-significant vs. 5 of 24 for methanotrophs).

**Figure 6:**
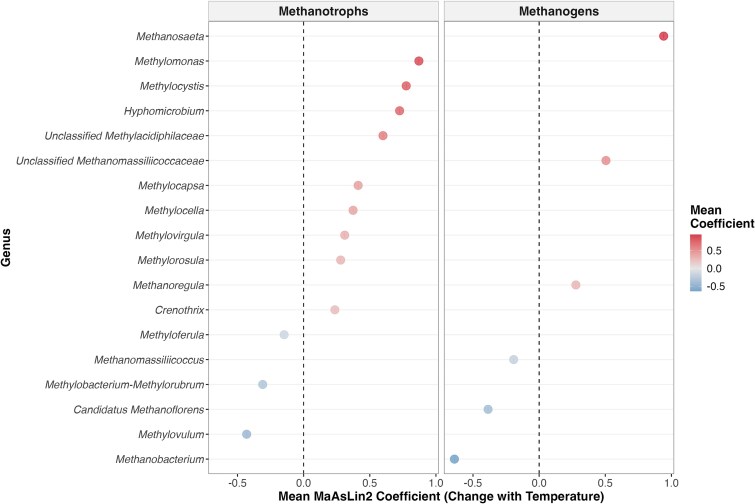
MaAsLin2 temperature coefficients for putative methane-cycling genera in porewater. Points represent the mean MaAsLin2 model coefficient for each genus significantly associated (*P* < .05) with measured temperature at 50 cm depth. Samples included four depths (25, 50, 100, 200 cm), all porewater years (2017, 2018, 2019, 2020, 2021), and all treatment enclosures. Genera are ordered by coefficient magnitude within each panel. Point color indicates the coefficient’s direction and magnitude, scaled from blue (negative, depleted with warming) through gray (neutral) to red (positive, enriched with warming). The dashed vertical line marks a coefficient of zero (no temperature effect).

MaAsLin2 analysis of genus-level temperature responses in porewater (2017–2021) further resolved these patterns (Fig. 6). Methanotrophic genera were predominantly enriched under warming (9 of 12 genera), with strong positive responses for *Methylomonas* (coef. = 0.87), *Methylocystis* (0.78), and *Hyphomicrobium* (0.73) spanning both Type I and Type II lineages. Methanogenic responses were more variable, but notably, the acetoclastic *Methanosaeta* showed the largest positive temperature coefficient among CH_4_-cycling taxa (0.94), while hydrogenotrophic genera, including *Methanobacterium* (−0.64) and Ca. *Methanoflorens* (−0.39) declined with warming.

Finally, total methanotroph and methanogen relative abundances showed statistically significant but opposing relationships with the porewater CO_2_:CH_4_ ratio across all depths and years (Supplemental Fig. 7): methanotroph abundance decreased with increasing CO_2_:CH_4_ (adj. R^2^ = 0.026, *P* < .001), while methanogen abundance increased (adj. R^2^ = 0.037, *P* < .001), consistent with the expected coupling between community composition and in situ gas dynamics.

## Discussion

Analysis of 812 amplicon-sequenced samples identified over 33 000 unique ASVs, with habitat identity (peat vs. porewater) emerging as the primary driver of microbial community composition, underscoring strong niche differentiation between these environments. Porewater assemblages exhibited stronger and more consistent compositional responses to warming and elevated CO_2_ across depths and years, with temperature treatment explaining a larger unique fraction of community variation in porewater than peat, positioning porewater microbiomes as particularly sensitive indicators of climate-driven ecosystem change. Within CH_4_-cycling communities, habitat partitioning was equally pronounced: methanotrophs were more abundant and taxonomically diverse in porewater, with abundance increasing with depth, whereas methanogens were concentrated in deeper peat horizons. Warming drove significant compositional shifts in porewater methanotroph communities across multiple depths and years, while methanogen responses were more variable, suggesting the two guilds differ in thermal sensitivity and that warming may shift the balance between CH_4_ production and oxidation in habitat-specific ways.

### Habitat dictates microbial diversity

The absence of any significant relationship between temperature and alpha diversity in peat (Supplemental Fig. 1) suggests that peat-associated communities are compositionally resistant to warming, likely reflecting the physical protection afforded by diffusion-limited microhabitats, nutrient retention, and biofilm formation in the solid-phase matrix [50, 51]. In contrast, the consistent decline in ASV richness with temperature across all porewater depths and the particularly strong Shannon diversity response at 25 and 50 cm indicate that warming exerts a selective filtering effect on porewater communities that is absent in peat. At shallow depths, reductions in both richness and evenness suggest competitive exclusion near the acrotelm lower boundary, where rhizosphere oxygen inputs and temperature sensitivity are greatest. At deeper depths, warming reduced richness without affecting evenness, consistent with the selective loss of rarer taxa while dominant community structure remained intact.

Microbial abundance responses to warming were also habitat- and depth-dependent (Supplemental Fig. 2). Bacterial abundance at 25 cm increased significantly with temperature in both habitats, suggesting shared surface-level thermal stimulation, but deeper responses diverged: warming-associated increases in bacterial abundance at 200 cm and archaeal abundance at 50 cm were detected only in peat, while the only significant effect on the bacterial:archaeal ratio occurred in porewater at 100 cm. Together, these patterns reinforce that peat and porewater microbiomes respond to warming through fundamentally different ecological processes, reflecting differences in habitat stability, substrate dynamics, and community turnover.

### Peat and porewater represent distinct microbial communities

The dbRDA analysis revealed that habitat and depth were the dominant factors shaping microbial community composition, with habitat exerting the most decisive influence (Fig. 1). This aligns with previous studies [31, 52] and reflects the distinct chemical and physical properties of peat and porewater, whose nutrient dynamics show little overlap despite continuous hydrological connectivity [53]. Analogous patterns have been observed in aquifer systems, where attached and free-living communities differ in abundance, diversity, and function even under shared environmental conditions [54–58].

Depth also represented a strong selective force shaping microbial community composition (Fig. 1), reflecting the stratified nature of peatland biogeochemistry. With increasing depth, oxygen availability declines, and labile organic compounds are replaced by more recalcitrant compounds, such as aromatics [29, 30, 59]. Previous research has demonstrated a close link between this shift in organic matter quality and the depth-driven stratification of microbial communities in peat [26, 28]. Here, we see that depth exerts a similar influence on the structuring of microbial communities within porewater.

In contrast, temperature and year had comparatively modest effects on microbial community composition (Fig. 1). Although statistically significant, together they explained only 1.40% of the community variance. However, when the analysis was restricted to porewater samples, temperature alone accounted for a larger proportion of variance (2.70%), highlighting the greater sensitivity of porewater microbial communities to warming.

The observed differences in microbial community composition between ambient peat and porewater samples suggest that substrate availability, terminal electron acceptors, and oxygen dynamics shape microbial niches (Fig. 2). Key functional guilds showed distinct spatial distributions: canonical aerobic methanotrophs, *Methylobacter, Methylomonas,* and *Crenothrix*, and the putative methanogen *Methanoregula* were predominantly detected in porewater, whereas sulfate reducers (*Desulfobacca, Syntrophobacter*) were more abundant in peat, reflecting greater anaerobiosis and reliance on solid-phase carbon. The prevalence of methanotrophs in porewater suggests a more active aerobic community, consistent with higher per-cell oxygen consumption in free-living versus attached microbes [60]. Warming-induced increases in plant-derived substrates, including root exudates and decomposing Sphagnum litter, likely drive these patterns, differentially influencing porewater and peat microbial communities [30, 61].

Porewater Communities are More Sensitive to Climate Drivers Than Peat Communities.

Microbial responses to warming and atmospheric CO₂ enrichment differed markedly between peat and porewater, with porewater communities showing greater sensitivity and a broader diversity of responsive taxa (Supplemental Fig. 3; Fig. 4). Free-living porewater communities are directly exposed to fluctuating DOM and carbon substrates and can respond rapidly to environmental perturbations, whereas particle-bound peat communities are embedded within a structurally complex, carbon-rich matrix that may buffer against short-term compositional change [13, 29, 62–64]. Structured soil and sediment matrices have been shown to exhibit slower extracellular DNA turnover than freshwater and marine environments, so peat may also harbor relic DNA or dormant cells that mask treatment effects [65]. Consistent with this, some phyla (e.g. Thermoplasmatota) responded exclusively in porewater, while no phyla responded uniquely in peat.

Warming significantly altered archaeal and bacterial groups in porewater spanning diverse metabolic guilds, including sulfur cycling (Desulfobacterota, Pseudomonadota), fermentation (Firmicutes, Bacteroidota, Elusimicrobiota, Spirochaetota), facultative heterotrophy (Actinobacteriota), and nitrification (Nitrospirota, Planctomycetota) (Fig. 4). Multiple archaeal lineages abundant in porewater but largely absent in peat declined significantly with warming (Fig. 4), consistent with reported reductions in peatland archaeal diversity under elevated temperatures [66]. Despite this, total archaeal abundance remained stable across warming treatments in porewater (Supplemental Fig. 2), suggesting that warming-induced increases in porewater CH₄ are driven by heightened methanogen activity rather than population growth, consistent with prior SPRUCE findings [19, 34] and the energetic constraints of methanogenesis under cold, acidic, anaerobic conditions [67, 68]. The broad depletion of specific archaeal phyla under warming, including Crenarchaeota, Thermoplasmatota, and Micrarchaeota in porewater and Crenarchaeota in peat, likely reflects the dominance of non-methanogenic lineages within these phyla, many associated with ammonia oxidation (Crenarchaeota) or parasitic lifestyles (Micrarchaeota, Nanoarchaeota), whose ecological roles may be undermined by warming-induced shifts in nitrogen cycling or host community composition. Conversely, the enrichment of Hydrogenedentes under warming in porewater may reflect a role in syntrophic hydrogen metabolism that becomes more favorable as fermentation rates increase, though this remains speculative.

Elevated CO₂ induced fewer community shifts than warming, with significant changes in 18 phyla in porewater and 10 in peat (Fig. 4). Peat-specific responses were limited to Nitrospirota and Planctomycetota, both associated with nitrogen or sulfur cycling under oligotrophic conditions [69, 70], while porewater displayed a broader response spanning metabolically versatile and syntrophic groups (e.g. Chloroflexi, Bacteroidota, Nanoarchaeota, Patescibacteria). Pseudomonadota, Verrucomicrobiota, and Acidobacteriota were consistently depleted under elevated CO₂ in both habitats. These phyla encompass many aerobic and facultative heterotrophs, and their shared depletion may reflect CO₂-driven shifts in redox conditions or pH that favor strictly anaerobic pathways, or changes in plant-derived substrate quality that disadvantage generalist heterotrophs. Elevated CO₂ enhances vascular plant productivity in peatlands [71, 72] and other wetlands [73, 74], increasing root biomass and delivery of fresh, labile substrates into the peat profile [24]. In the deep peat (up to 200 cm), where terminal electron acceptors are severely limited, increased labile carbon inputs would be expected to stimulate anaerobic decomposition rather than aerobic pathways. Consistent with this shift toward anaerobic metabolism, fermentative phyla such as Bacteroidota and Fibrobacterota increased significantly in porewater under elevated CO₂ (Fig. 4).

Variance partitioning and dbRDA results further clarify how environmental drivers structure communities differently across habitats (Supplemental Fig. 4; Supplemental Table 2). In peat, depth and DOC dominated community variation, reflecting the tight coupling between vertical position and organic matter quality. Here, DOC likely indexes decomposition-driven gradients in substrate recalcitrance rather than fresh plant inputs, and solid-phase communities appear buffered against warming by physical and chemical protection within the peat matrix. This is consistent with the small unique variance attributable to temperature (1.07%) in peat. In porewater, temperature’s larger unique effect (4.70%) and the substantial variance shared among DOC, CO_2_, and CH_4_ (6.93%) suggest that warming restructures communities partly through covarying biogeochemistry rather than direct thermal effects alone. DOC’s significant independent effect in both habitats supports a central role for labile carbon in mediating community composition. Warming-induced shifts in vegetation at SPRUCE, including the rapid expansion of vascular plants that enhance labile DOM inputs [28, 30], likely underpin these biogeochemical linkages, with elevated CO₂-enhanced gross primary production [24] amplifying belowground carbon allocation and further altering porewater chemistry [75, 76]. Radiocarbon studies confirm that porewater DOM is younger and more labile than bulk peat carbon at SPRUCE, with microbial respiration products (CO₂ and CH₄) isotopically resembling porewater DOM [17], underscoring the bioavailability of aqueous carbon substrates fueling these community shifts. While previous work documented enhanced greenhouse gas production without corresponding shifts in peat communities [30], we demonstrate that porewater microbiomes respond in parallel with increased emissions, underscoring porewater as a sensitive indicator of ecosystem responses to climate drivers.

### Porewater methane cycling taxa respond to warming

CH_4_-cycling taxa exhibited distinct distributional patterns across peat and porewater that reflect the contrasting biogeochemical conditions of each habitat (Supplemental Fig. 5). Putative methanotrophs were significantly more abundant in porewater below the surface, with relative abundance increasing with depth. In peat, methanotrophs showed the opposite pattern, declining steadily below 50 cm. Methanogens were more abundant in porewater at shallow depths (25–50 cm), while in peat their abundance increased with depth (Supplemental Fig. 5).

Warming responses were similarly compartmentalized between habitats and between CH_4_ producers and consumers (Fig. 5). Methanotroph relative abundance increased significantly with temperature in porewater across most year × depth combinations, with particularly consistent responses at 50 and 100 cm. In contrast, peat methanotrophs more commonly declined with warming. Methanogens exhibited predominantly negative temperature responses in both habitats, though their sensitivity was more variable, with roughly half of the methanogen models being nonsignificant, compared with only a quarter of methanotroph models.

Temperature-driven increases in porewater methanotrophs to 200 cm depth (Fig. 5) parallel earlier findings at SPRUCE, which show warming-induced increases in CO₂ concentrations across the peat profile [30]. The unexpected persistence and increase in specific methanotroph taxa at depth, despite declining oxygen availability, may indicate either anaerobic CH_4_ oxidation [26, 77] or oxygen delivery via porewater transport [78]. Combined with the stability of total archaeal abundance under warming (Supplemental Fig. 2), these results suggest that warming shifts the balance of CH_4_ cycling in porewater by simultaneously stimulating CH_4_ consumption and reducing the relative abundance, though not necessarily the activity, of CH_4_ producers.

The distributions of putative methanotrophic and methanogenic genera reflected strong trends with habitat and depth (Supplemental Fig. 6). Among methanotrophs, *Methylocystis* dominated peat across all depths, with the highest abundance in shallow horizons (25–50 cm) and a pronounced decline below 100 cm, while in porewater, it peaked at 100 cm. *Methylobacter* and *Crenothrix* were effectively absent from peat but increased substantially with depth in porewater, consistent with growth under oxygen-limited, stratified conditions [79]. Notably, *Methylobacter* and members of the *Methylomirabilaceae* have also been shown to employ alternative electron acceptors under low-oxygen conditions [80, 81], indicating broader functional flexibility than their habitat distributions alone would suggest. Sh765B-TzT-35 (*Methylomirabilaceae*) accumulated in deep peat while remaining rare in porewater, consistent with reported capacity for anaerobic CH_4_ oxidation using nitrite or metals as electron acceptors [82–84]. Methanogen distributions were equally habitat-structured. Ca. Methanoflorens was the dominant methanogen in both habitats, but with contrasting depth profiles: in peat it peaked at 100 cm, whereas in porewater it declined sharply below 50 cm, suggesting a depth-related shift in community structure that was not mirrored in the solid matrix. *Methanoregula* increased with depth in both habitats, most steeply in porewater, whereas unclassified *Methanomicrobiaceae* were restricted almost entirely to deep peat and absent from porewater. It should be noted that these genus-level patterns reflect distributional trends in 16S rRNA amplicon data and are not directly supported by functional gene or metabolic data; species within the same genus can vary substantially in their physiology, so these interpretations are necessarily provisional.

An analysis across all porewater depths and years (2017–2021) revealed divergent temperature responses between putative CH_4_-cycling guilds (Fig. 6). The majority of methanotrophs were enriched under warming, and the strongest positive responses were observed for *Methylomonas, Methylocystis*, and *Hyphomicrobium. Methylocystis* enrichment may reflect its tolerance of fluctuating oxygen and CH_4_ concentrations [85–87], while *Methylomonas* may benefit from its capacity to use alternative electron acceptors under oxygen limitation [77, 85]. The increased abundance of *Hyphomicrobium*, a facultative methylotroph, may suggest that warming enhances the accumulation of methanol and other C1 compounds through increased plant and microbial turnover [88, 89]. The obligate methanotroph *Methyloferula* was depleted with warming, consistent with the vulnerability of niche specialists to shifting redox and substrate regimes [90–92].

Methanogens exhibited more muted and evenly split responses to warming (Fig. 6). The most notable shift was toward acetoclastic over hydrogenotrophic pathways: *Methanosaeta*, a high-affinity acetate utilizer, showed the largest positive coefficient among all CH_4_-cycling taxa [93, 94], while hydrogenotrophic *Methanobacterium* and Ca. Methanoflorens declined with warming. *Methanoregula* was a notable exception among hydrogenotrophs, increasing modestly with temperature. Collectively, these patterns suggest warming restructures methanogen communities by favoring acetoclastic over hydrogenotrophic pathways, consistent with increased acetate availability reported under warming in peatland systems [26, 27].

The opposing associations between methanotroph and methanogen relative abundance and the porewater CO₂:CH₄ ratio provide additional geochemical context for these community patterns (Supplemental Fig. 7). A high CO₂:CH₄ ratio reflects conditions unfavorable for methanogenesis: reduced substrate availability, active CH_4_ consumption, or both. The positive association between methanogen abundance and this ratio may appear counterintuitive, but it is consistent with a scenario in which methanogen enrichment occurs under substrate-limited conditions that drive CH_4_ production deeper into the peat column, where CO_2_ from heterotrophic decomposition accumulates while CH_4_ is consumed before reaching shallow porewater. The decline in methanotroph abundance with increasing CO_2_:CH_4_ ratio likely reflects reduced CH_4_ availability, which constrains growth. Although explained variance was modest (adj. R^2^ = 0.026–0.037), significance across the full dataset confirms that porewater chemistry is a consistent, albeit partial, predictor of CH_4_-cycling community composition. The low R^2^ values are expected due to the combined influence of depth, temperature, and organic carbon.

Together, these results highlight the spatial partitioning of CH_4_-cycling guilds across peat and porewater habitats and demonstrate that warming selects for metabolically flexible taxa within both guilds while disadvantaging niche specialists, underscoring the importance of incorporating microbial community dynamics into peatland carbon models.

## Conclusions

Peat and porewater harbor distinct microbial communities that differ in composition, diversity, and response to climate drivers. Habitat partitioning was the dominant factor shaping community structure across years and depths, reflecting the physicochemical contrast between the solid organic matrix and the dissolved phase and the distinct selective pressures each imposes. Within this framework, porewater communities consistently responded more strongly to warming than peat, with a greater number and magnitude of temperature-associated compositional shifts across multiple years. These habitat-specific responses carry direct implications for peatland carbon cycling. Warming-associated increases in porewater methanotroph abundance, coupled with declining methanogen abundance in both habitats, indicate that CH_4_ cycling is more dynamic and climate-responsive than previously appreciated. If warming differentially shifts the balance between CH_4_ production and oxidation across habitats, net CH_4_ emissions may reflect community-level reorganization that bulk flux measurements cannot resolve. The added CO_2_ sensitivity of porewater communities further suggests that climate-driven changes in dissolved substrate availability could cascade through the aqueous phase in ways the peat-attached community does not register.

Resolving the functional and metabolic potential of porewater communities, and the contrast between attached and free-living populations, will be critical for understanding climate–peatland feedbacks. Because porewater sampling is non-destructive and repeatable, it offers a scalable approach for long-term microbial monitoring that destructive peat coring cannot match. Given that peatlands store roughly one-third of global soil carbon and are a major natural source of CH_4_, prioritizing habitats that respond earlier and more strongly to climate forcing is essential for improving predictive models. Future work should target the metabolic potential of porewater communities via metagenomics and stable-isotope approaches, and characterize how substrate availability, terminal electron acceptor dynamics, and oxygen gradients interact across the peat–porewater interface to mediate the responses documented here.

## Supplementary Material

Supplementary_material_ycag164

## Data Availability

The raw 16S rRNA gene sequences generated during this study are available in the NCBI BioProject database under accession number PRJNA1327265 (http://ncbi.nlm.nih.gov/bioproject). The SPRUCE environmental datasets analyzed during this study are publicly available in the SPRUCE long-term data repository: 50 cm peat temperature measurements (http://doi.org/10.3334/CDIAC/spruce.032) and porewater geochemistry data (http://doi.org/10.3334/CDIAC/spruce.028 and http://doi.org/10.25581/spruce.083/1647173).
