# Supplementary material for: Porewater microbial community dynamics act as an indicator of northern peatland ecosystem change in response to climate drivers
Source: ISME Commun. 2026 Jun 15;6(1):ycag164. doi: 10.1093/ismeco/ycag164 (PMC13374860; doi:10.1093/ismeco/ycag164)
Supplement: Supplementary_material_ycag164 [file supplementary_material_ycag164.zip › Supplemental_Figure_Captions_Updated.docx]

Supplemental Figure 1. **Alpha diversity trends with temperature in peat and porewater communities.** Data are from rarefied 16S rRNA gene ASV communities collected from three years (2017, 2018, 2019), four depths (25, 50, 100, 200 cm), and all treatment enclosures. Points are colored by depth and arranged by habitat (columns) and depth (rows). Dashed regression lines with 95% confidence intervals are shown only for habitat × depth combinations with significant linear relationships (p < 0.05); R² and p-values are annotated on significant panels.

Supplemental Figure 2. **Quantitative PCR (qPCR) abundance of peat and porewater samples with temperature across habitats and depths.** Panels show (A) bacterial SSU rRNA gene copies per mL, (B) archaeal SSU rRNA gene copies per mL, and (C) the bacteria:archaea ratio, each plotted against measured peat temperature at 50 cm depth. Samples were collected only in 2019, spanned four depths (25, 50, 100, 200 cm), and included all treatment enclosures. Samples are faceted by depth (rows) and habitat (columns), and their abundance values are displayed on a log₁₀ scale to accommodate the range of gene copy abundances across samples. Dashed black lines with gray confidence bands indicate significant linear regressions (p < 0.05) of log₁₀-transformed values with temperature, fit independently within each habitat × depth combination. R² and p-values are annotated for significant models only. Points are colored by habitat: blue for porewater, tan for peat.

Supplemental Figure 3. **Number of peat and porewater ASVs significantly associated with temperature and CO_2_ identified by MaAsLin2 across depths.** Bar charts show the count of significant ASVs (p < 0.05) identified by MaAsLin2 for measured temperature at 50 cm depth (top row) and CO₂ treatment concentrations (bottom row). Samples include three years (2017, 2018, 2019), four depths (25, 50, 100, 200 cm), and all treatment enclosures. Data are from genus-level analyses, faceted by depth, and colored by habitat: blue for porewater, tan for peat.

Supplemental Figure 4. **Distance-based redundancy analysis (db-RDA) of microbial community composition constrained by environmental variables across peat and porewater habitats.** Ordination was performed using Bray–Curtis dissimilarity, with six constraining variables: measured temperature at 50 cm depth, dissolved organic carbon, porewater CH₄, porewater CO₂, and depth (cm). Points are colored by DOC concentration, and biplot arrows indicate the direction and relative magnitude of each environmental variable's loading on the constrained axes. Axis labels report the percentage of total (constrained + unconstrained) variation explained by each db-RDA axis. Samples include three years (2017, 2018, 2019), four depths (25, 50, 100, 200 cm), and all treatment enclosures.

Supplemental Figure 5. **Putative methanotroph and methanogen relative abundance by habitat and depth.** Boxplots show total relative abundance (%) of methanotrophs (top row) and methanogens (bottom row) in peat and porewater communities, faceted by depth. Data are from genus-level relative abundance estimates of samples collected from three years (2017, 2018, 2019), four depths (25, 50, 100, 200 cm), and all treatment enclosures. Individual samples are shown as jittered points. Significance brackets indicate Wilcoxon rank-sum tests with BH correction (*p < 0.05, **p < 0.01, ***p < 0.001, ****p < 0.0001).

Supplemental Figure 6. **Genus-level relative abundance of putative methanotrophs and methanogens in peat and porewater.** Temperature treatment-averaged relative abundances of the most abundant (A) methanotrophs and (B) methanogens, split into high-abundance (≥ 1% for methanotrophs, ≥ 0.2% for methanogens) and low-abundance panels to prevent visual compression of rare taxa. Samples include three years (2017, 2018, 2019), four depths (25, 50, 100, 200 cm), and all treatment enclosures. Bars are faceted by habitat and colored by Genus.

Supplemental Figure 7. **Putative methane-cycling taxa relative abundance versus porewater CO₂:CH₄ molar ratio.** Total relative abundance (%) of methanotrophs and methanogens plotted against the porewater CO₂:CH₄ molar. Panels show points colored by (A) depth and (B) measured temperature. Dashed black regression lines with 95% confidence intervals represent overall linear fits, with R² and p-values annotated per functional group. Samples included in this analysis were from five years (2017, 2018, 2019, 2020, 2021), four depths (25, 50, 100, 200 cm), and all treatment enclosures.
