# Supplementary material for: Porewater microbial community dynamics act as an indicator of northern peatland ecosystem change in response to climate drivers
Source: ISME Commun. 2026 Jun 15;6(1):ycag164. doi: 10.1093/ismeco/ycag164 (PMC13374860; doi:10.1093/ismeco/ycag164)
Supplement: Supplementary_material_ycag164 [file supplementary_material_ycag164.zip › Supplemental_Methods.docx]

**Supplemental Methods**

**Site Infrastructure**

The S1 bog (418 m elevation) is a waterlogged ombrotrophic peatland with deep peat deposits typical of northern peatlands. SPRUCE enclosures are octagonal, 7 m tall × 12.8 m diameter (114.8 m²). Whole-ecosystem warming is delivered by propane-fired heat exchangers that distribute warmed air through wall diffusers at 1 m height, supplemented by subsurface resistance heaters at 3 m depth. Elevated CO_2_ is injected into the air handling ductwork to test how rising atmospheric CO_2_ modulates ecosystem responses to warming; the +500 ppm target represents an upper-bound projection for end-of-21st-century atmospheric concentrations.

**Porewater Sample Collection Details**

Porewater was collected from permanently installed PVC piezometers (2.5 cm diameter, screen-mesh bottom) emplaced at multiple depths below the peat hollow surface (10, 25, 50, 75, 100, 125, 150, 175, and 200 cm); for this study we sampled the 25, 50, 100, and 200 cm horizons during peak growing season (late July–early August) across five years (2017–2021). Prior to each sampling, piezometers were pumped dry and allowed to recharge for 12 h, then sampled by peristaltic pump connected to a syringe. Collected volumes scaled with depth: 50 mL at 25 and 50 cm, 100 mL at 100 cm, and 150 mL at 200 cm. Samples were filtered through 0.2 μm sterile filters within 1 h of collection and stored on dry ice.

**qPCR Primers and Volumetric Correction**

Reactions used primers 331F (5′-CCTACGGGAGGCAGCAGT) / 518R (5′-ATTACCGCGGCTGCTG) for bacteria and Arch787F (5′-ATTAGATACCCSBGTAGTCC) / Arch1059R (5′-GCCATGCACCWCCTC) for archaea. The volume of wet peat (V_sw) used for DNA extraction was calculated as:

$$r_{w}=\frac{m_{d}(1+f)}{V_{w}}(1)$$

$$V_{sw}=\frac{m_{s}}{r_{w}}(2)$$

where m_s is the mass of wet peat before DNA extraction, m_d is the dry mass of the composited peat sample per depth increment, f is the gravimetric water content, and V_w is the total peat volume sampled per depth increment.

**Amplicon Primer Sets**

Peat samples from 2017–2018 (UIC) were amplified with CS1_515F (5′-ACACTGACGACATGGTTCTACA_GTGCCAGCMGCCGCGGTAA-3′) and CS2_806R (5′-TACGGTAGCAGAGACTTGGTCT_GGACTACHVGGGTWTCTAAT-3′). Samples from 2019–2021 (Georgia Tech) were amplified using 515F-Y (5′-GTGYCAGCMGCCGCGGTAA-3′) and 806R-Apprill (5′-GGACTACNVGGGTWTCTAAT-3′) with overhang adapters. Libraries were sequenced on an Illumina MiSeq 2000 using a 500-cycle v2 kit.

**Alpha Diversity Modeling**

To assess depth- and habitat-specific diversity–temperature relationships, OLS regressions were fit independently for each metric × habitat × depth combination, with regression lines, R², and p-values shown only where p < 0.05.

**dbRDA Specification**

For the primary dbRDA, two-way interactions improved fit (adj. R² = 0.342 vs. 0.270 for the additive model), but because main effects were consistent, we present the additive model. For the secondary dbRDA, depth × biogeochemistry interactions did not improve fit (0.239 vs. 0.217). VIF was < 10 for all predictors, and significance was evaluated with 999 free permutations supplemented by restricted permutations blocking by plot.

**MaAsLin2 Specification**

Stratification by habitat × depth addressed non-independence from repeated plot sampling. Plot was excluded as a random effect to avoid absorbing between-plot variance confounded with treatment. Inputs were pre-normalized relative abundances (normalization = "NONE"); Benjamini–Hochberg FDR correction was applied within each model with thresholds q < 0.25, p < 0.05, minimum prevalence 0.05, and minimum abundance 0.001.

**Methane-Cycling Taxon Regressions**

For temperature responses (**Figure 5**), relative abundance at the measured 50 cm temperature was fit for each functional group × habitat × depth × year (2017–2019), restricted to combinations with n ≥ 5. For dissolved gas relationships (**Supplemental Figure 7**), total relative abundance was regressed against porewater CH_4_ and CO_2_ across all depths and years. Regression lines with 95% confidence intervals are shown only for significant models (p < 0.05) and are annotated with R² and p-values.
