# Supplementary figures and images for: Porewater microbial community dynamics act as an indicator of northern peatland ecosystem change in response to climate drivers
Source: ISME Commun. 2026 Jun 15;6(1):ycag164. doi: 10.1093/ismeco/ycag164 (PMC13374860; doi:10.1093/ismeco/ycag164)

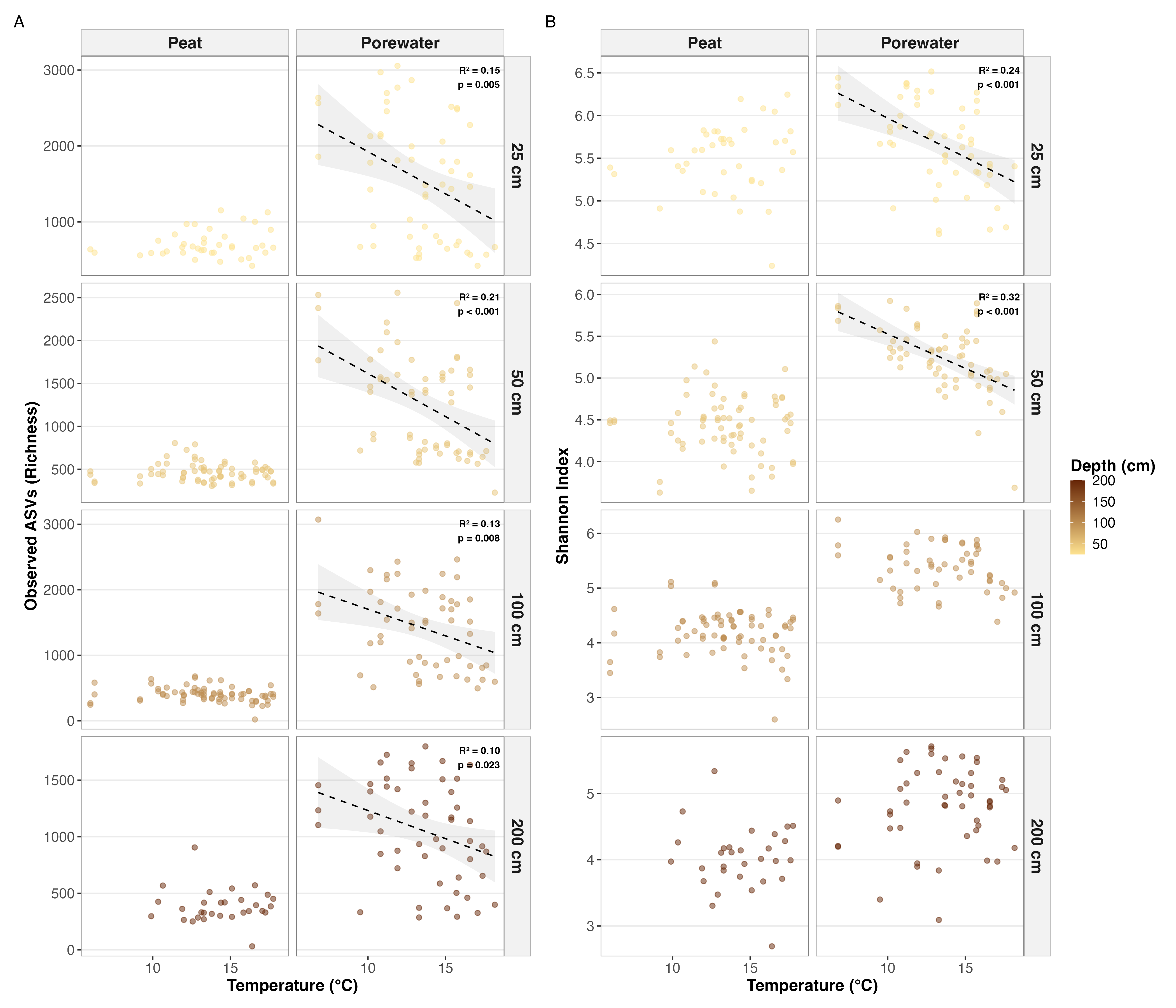

Supplement: Supplementary_material_ycag164 [file supplementary_material_ycag164.zip › Supplemental_FIgure_1.tiff]

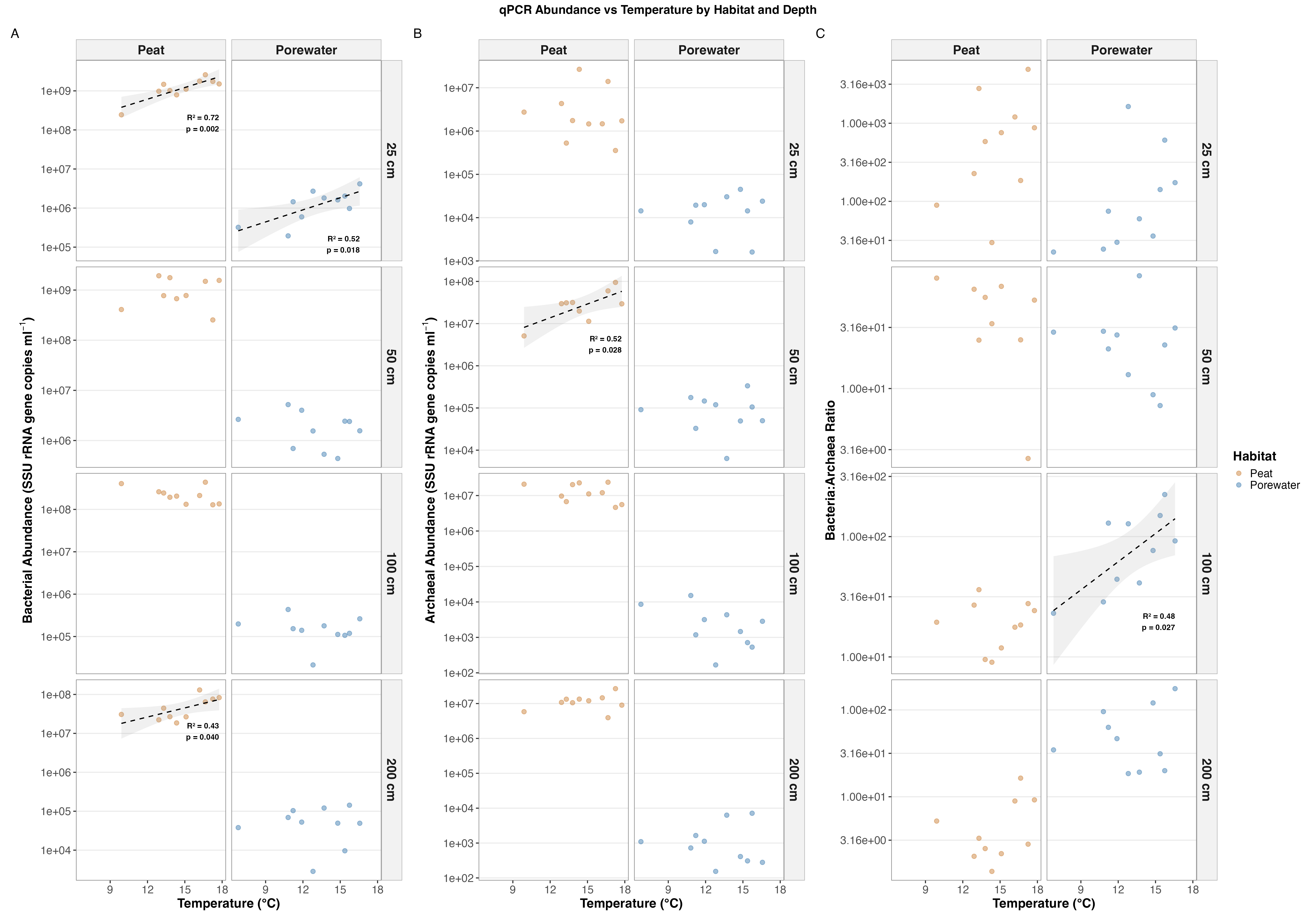

Supplement: Supplementary_material_ycag164 [file supplementary_material_ycag164.zip › Supplemental_Figure_2.tiff]

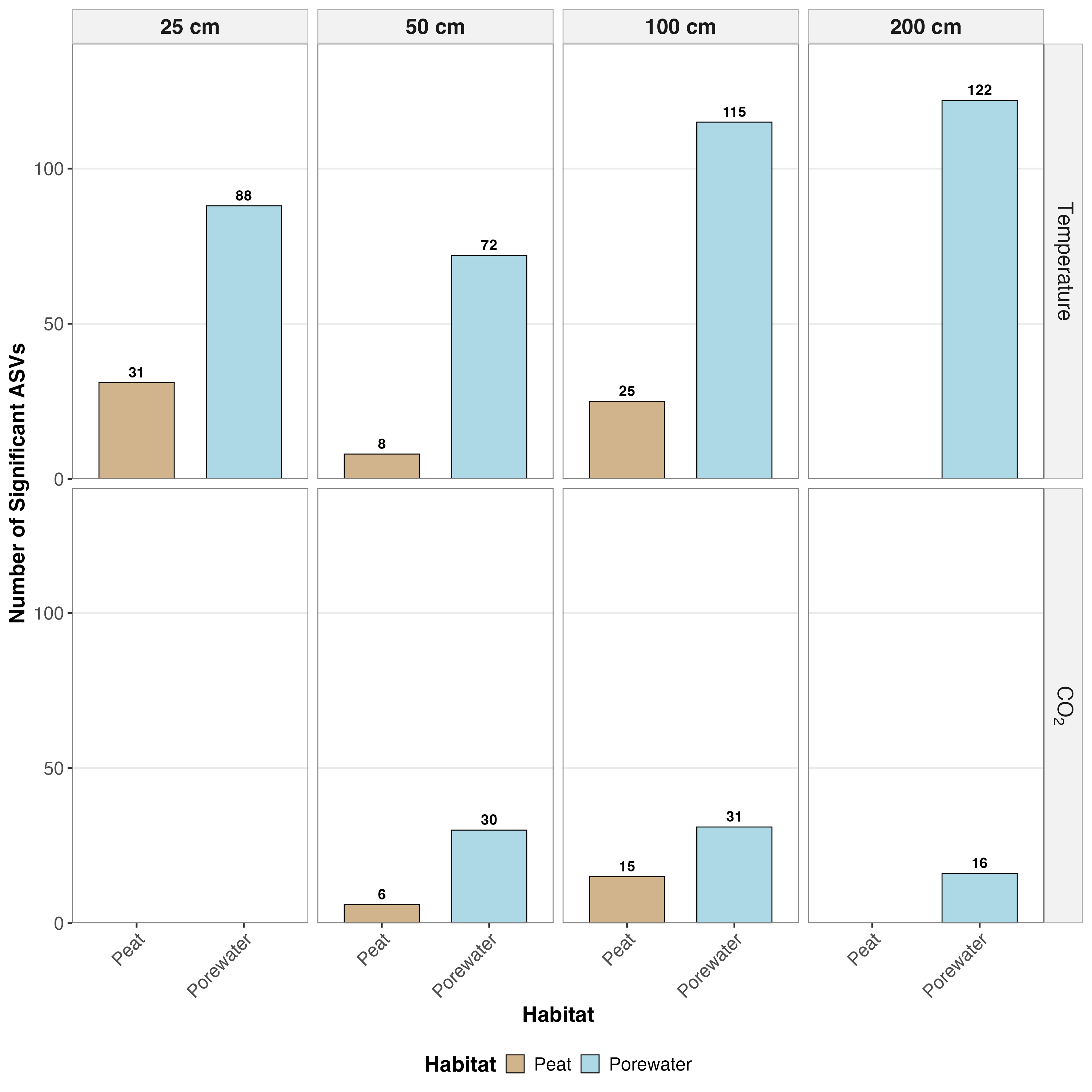

Supplement: Supplementary_material_ycag164 [file supplementary_material_ycag164.zip › Supplemental_Figure_3.tiff]

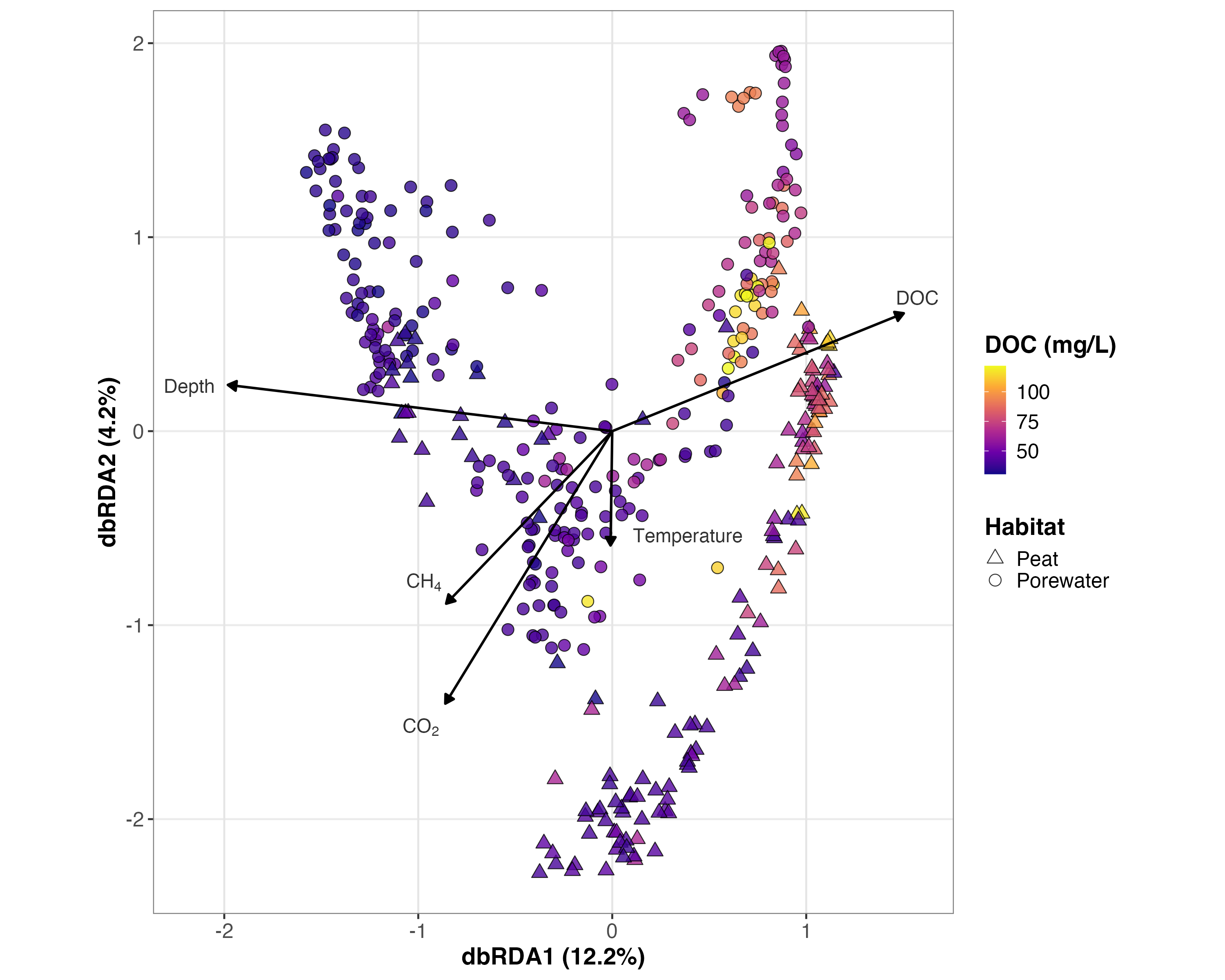

Supplement: Supplementary_material_ycag164 [file supplementary_material_ycag164.zip › Supplemental_Figure_4.tiff]

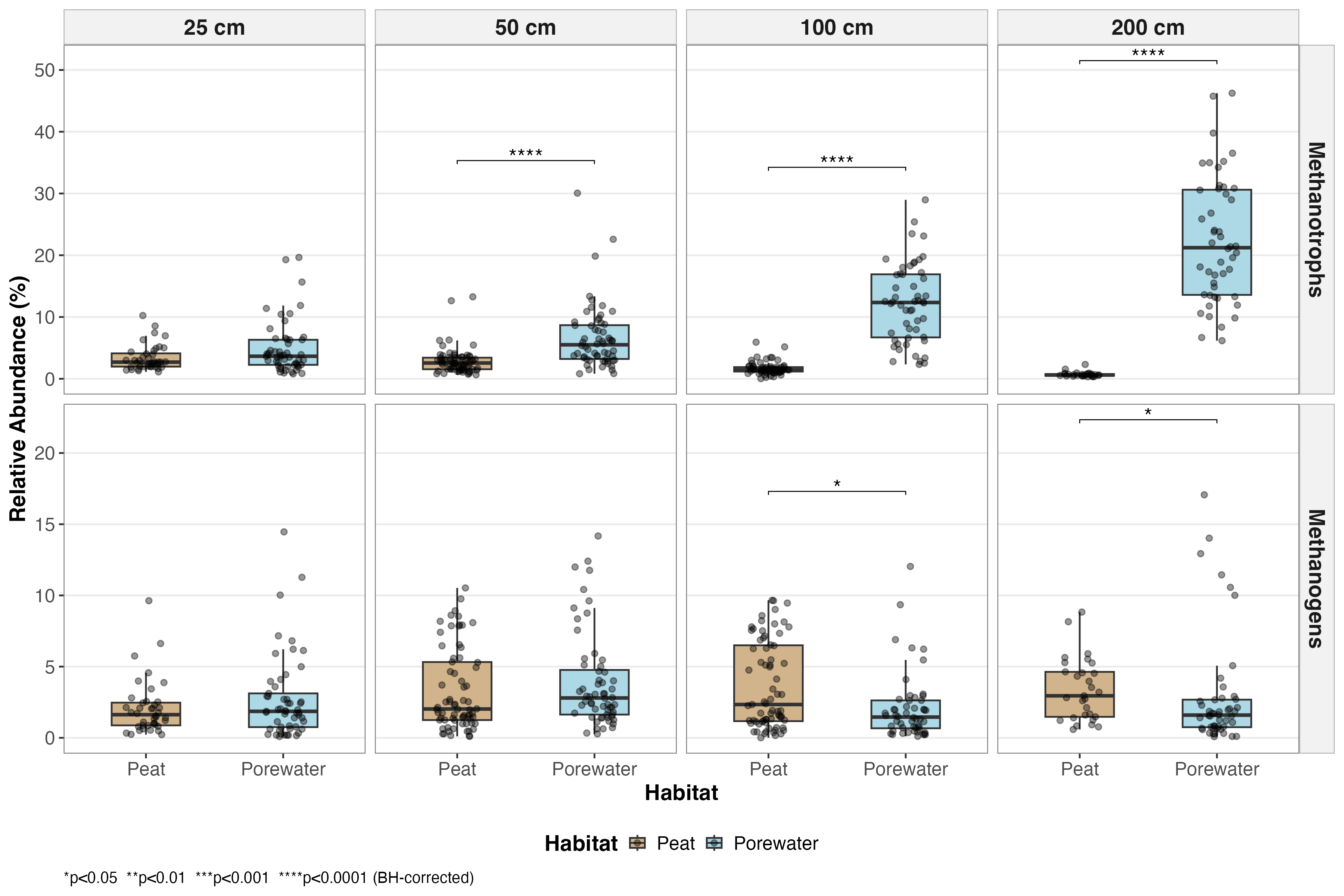

Supplement: Supplementary_material_ycag164 [file supplementary_material_ycag164.zip › Supplemental_Figure_5.tiff]

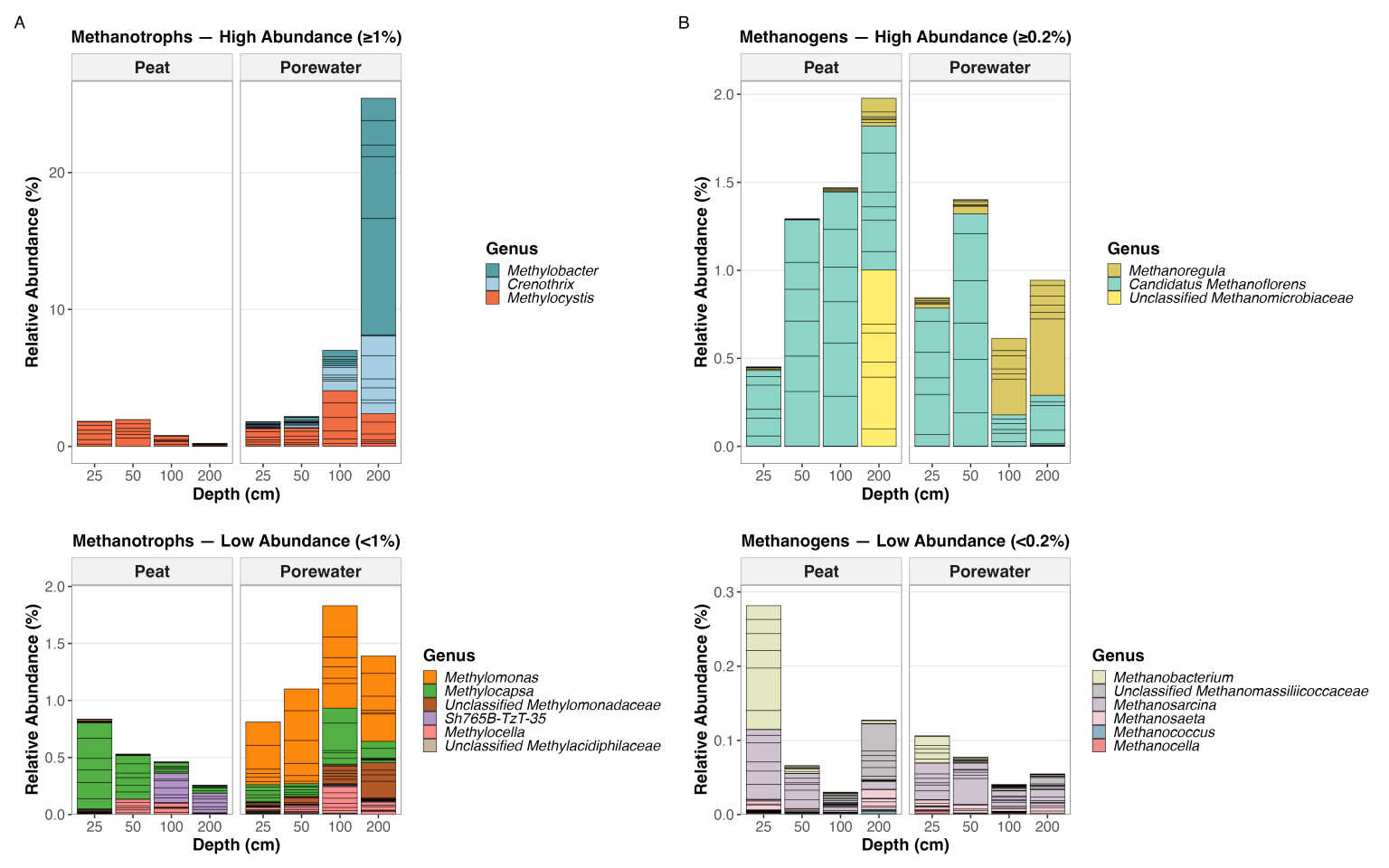

Supplement: Supplementary_material_ycag164 [file supplementary_material_ycag164.zip › Supplemental_Figure_6.tiff]

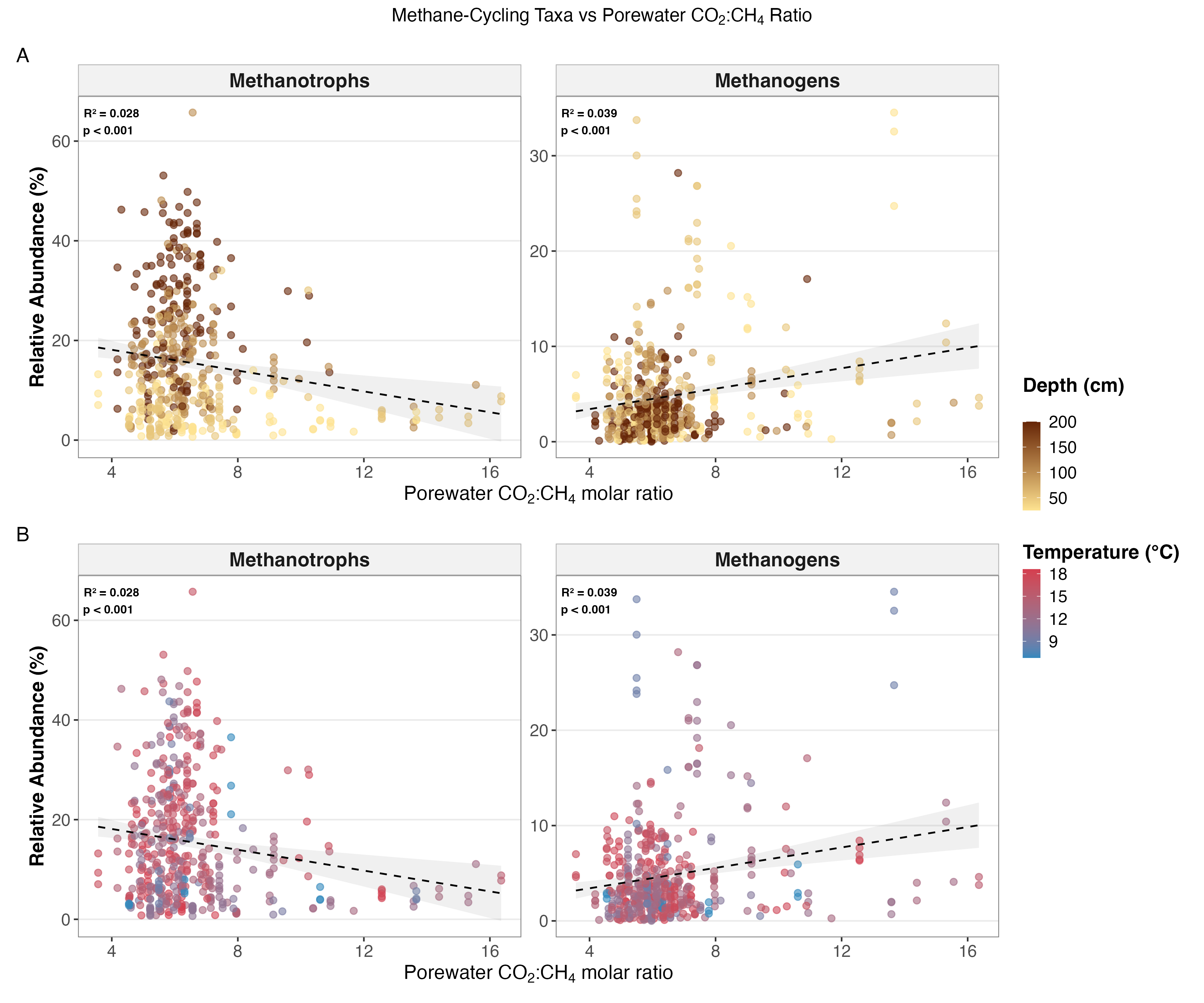

Supplement: Supplementary_material_ycag164 [file supplementary_material_ycag164.zip › Supplemental_Figure_7.tiff]
